# Attitudes of undergraduate medical students toward patients’ safety in Jordan: a multi-center cross-sectional study

**DOI:** 10.1186/s12909-023-04672-9

**Published:** 2023-09-22

**Authors:** Ibrahim Al-Sawalha, Nebras Jaloudi, Shaima’ Zaben, Rawan Hamamreh, Hala Awamleh, Sondos Al-Abbadi, Leen Abuzaid, Faisal Abu-Ekteish

**Affiliations:** 1 Faculty of Medicine, Jordan University of Science and Technology, Irbid, Jordan; 2 Faculty of Medicine, University of Jordan, Amman, Jordan; 3 Faculty of Medicine, The Hashemite University, Zarqa, Jordan; 4 Faculty of Medicine, Al-Balqa’ Applied University, As-Salt, Jordan; 5 Faculty of Medicine, Mutah University, Al-Karak, Jordan; 6 Faculty of Medicine, Yarmouk University, Irbid, Jordan; 7 Department of Pediatrics and Neonatology, Jordan University of Science and Technology, Irbid, Jordan

**Keywords:** Attitudes, Medical students, Patient safety, Medical errors, Jordan

## Abstract

**Background:**

Patient safety practices are crucial in healthcare as they aim to reduce harm, medical errors, and ensure favorable outcomes for patients. Therefore, this study aims to examine the attitudes towards patient safety among undergraduate medical students in Jordanian medical schools.

**Methods:**

A descriptive cross-sectional study was conducted among undergraduate medical students. Participants completed the Attitudes to Patient Safety Questionnaire- III (APSQ-III), which examines students’ attitudes in 26 items distributed in nine domains. Results are represented as mean ± standard deviation for all participants and subgroups.

**Results:**

Our study included 1226 medical students. They reported positive attitudes toward patient safety with a mean score of 4.9 (SD ± 0.65). Participants scored the highest score in “Working hours as error cause” followed by “Team functioning”. Gender, academic-year, and first-generation student status had a significant association with certain patient safety domains. Females scored significantly higher than males in four domains, while males scored higher in one domain. First-generation medical students had a significantly lower score for “Professional incompetence as error cause”. Interestingly, pre-clinical students recorded more positive attitudes in “Patient safety training received” and “Disclosure responsibility” domains.

**Conclusion:**

Undergraduate medical students in Jordan demonstrated positive attitudes towards patient safety concepts. Our study provides baseline data to improve current educational programs and enhance the patient safety culture among medical students. Additional studies are needed to delve into actual attitudes toward patient safety and to assess how educational programs contribute to the cultivation of this culture.

## Background

As defined by the World Health Organization (WHO), patient safety is a set of coordinated activities that aim to reduce avoidable harm and medical errors for patients undergoing healthcare [1]. Medical errors are unintentional actions or ones that ultimately do not achieve their expected outcome [2]. In the United States, medical errors are the third leading cause of death, accounting for 251,000 deaths annually [3]. Meanwhile, in developing countries the adverse events resulting from medical errors are greater than in developed countries [4]. A retrospective study by Wilson et al. assessed the frequency and nature of adverse events in patients in eight developing countries, including Jordan [5]. It was found that up to 18.4% of inpatient admissions were complicated by adverse events, and about 34% of those events occurred due to therapeutic errors in a relatively uncomplicated clinical situations.

Healthcare is classified as a “safety-critical industry” since errors and design failures lead to the loss of lives [6]. It is a combination of patients, complex systems, advanced technology, and fallible professionals. Since medical students are part of the healthcare system, it is of great importance to develop a culture of patient safety among them. Nevertheless, after patient safety education is received by medical students, studies routinely highlight the incompetence of medical students in reporting medical errors [7, 8]. They showed resistance and a lack of confidence in disclosing medical errors [8, 9]. Although reporting incidental errors improves patient safety by giving an opportunity to individual healthcare providers and organizations to learn from disclosed accidents [10, 11].

Medical school curricula have historically placed a strong emphasis on three main competencies: medical knowledge, technical skills, and judgment-clinical decision-making [12]. However, non-technical and professional competencies such as teamwork, leadership, human factors, and risk management were not usually taught. Recently, numerous accreditation bodies have acknowledged the urgent need for patient safety education for healthcare students. As a response, the WHO developed a patient safety curriculum guide for medical schools [13]. This guide aims to assist medical schools’ instructors in delivering patient safety education and faculties in elucidating patient safety knowledge and skills.

There is a growing interest in patient safety concepts among healthcare providers, reflecting the increasing recognition of their significance in improving healthcare outcomes and ensuring the well-being of patients. Al-Nawafleh et al. [14] studied patient safety cultures among healthcare providers in Jordanian hospitals. Healthcare providers included physicians, nurses, pharmacists, dieticians, physiotherapists, laboratory specialists, radiologists, and technicians. They demonstrated that 40% of participants reported at least one patient safety event in the 12 months preceding their study. Additionally, a recent study from Jordan [15], assessing medical errors among nurses and nursing student, have found that medical errors are highly prevalent, with more than 70% of nurses and nursing students have no training in reporting and preventing medical errors. As current medical students are the future healthcare providers, it is important to assess and address their knowledge, attitudes, and training regarding patient safety to ensure a proactive and effective approach in reporting and preventing medical errors [16].

Nowadays, patient safety concepts have been integrated into many medical schools’ curricula [17]. Patient safety courses are given either as traditional lectures, workshops, or other teaching methods. Clerkship directors in internal medicine at US and Canadian medical schools agreed that patient safety education should occur during medical school [18]. Studies assessing patient safety training interventions [19, 20] reported that receiving patient safety education is associated with positive behavioral changes among medical students, in addition to improvements in knowledge, skills, and attitudes. No study has assessed medical students’ attitudes toward patient safety concepts in Jordan. The findings of our study can provide baseline data for medical students’ attitudes and help in the future improvement of the current educational programs to promote a culture of patient safety and safe practices among medical students. Thus, the aim of this study is to explore patient safety attitudes among undergraduate medical students in all Jordanian medical schools.

## Methods

### Study design and settings

A cross-sectional descriptive study was conducted in January 2023 using a self-administered questionnaire. The target population was undergraduate medical students in all Jordanian medical schools: Jordan University of Science and Technology, University of Jordan, Yarmouk University, Al-Balqa’ Applied University, Hashemite University, and Mutah University. The academic program in the Jordanian medical schools is a total of six years. The first three years are the pre-clinical stage, while the last three years are the clinical stage. Participants were recruited through nonprobability convenience sampling. Eligibility criteria included being a current medical student at one of the Jordanian universities and having the willingness to participate in our study. Graduates, non-medical students, and medical students from non-Jordanian universities were excluded.

### Sample Size

We calculated the required sample size using the online Raosoft sample size calculator [21]. The recommended sample size, with 5% margin of error, 99% confidence level, 50% response rate and 20,000 population size, was 643. Correspondingly, our study sample size included 1226 medical students currently enrolled in Jordanian universities.

### Questionnaire design

A self-administered online questionnaire was constructed using Google Forms. The introduction page of the questionnaire explained the aims of the study and that participating in the study was voluntary. Participants were assured that their information is confidential and will be used only for the purpose of the study. Informed consent was obtained before they can proceed to the questions page. The questionnaire was posted on their Facebook groups and sent to targeted individuals using social media platforms.

Our questionnaire was divided into two categories: (a) participant demographics, and (b) attitudes to patient safety questionnaire III (APSQ-III). Participants’ demographic category included age, gender, current academic year, medical school and first-generation medical student status, defined as the first in their families to attend medical school [20]. Questions inquiring about current major, current academic year and medical school had several options to exclude individuals who do not fulfill our eligibility criteria. The APSQ-III was developed and validated by Carruthers et al. [22] with reliability coefficients from 0.64 to 0.82. It measures attitudes toward patients’ safety among medical students using 26 questions distributed in nine domains: patient safety training received (3 items), error reporting confidence (3 items), working hours as error cause (3 items), error inevitability (3 items), professional incompetence as error cause (4 items), disclosure responsibility (3 items), team functioning (2 items), patient involvement in reducing error (2 items), importance of patient safety in the curriculum (3 items). Responses to each question were rated on a Likert scale ranging from 1 (strongly disagree) to 7 (strongly agree). Higher scores indicated more affirmative responses to the corresponding items. Some items were reverse-coded as they indicated negative beliefs.

Positive responses were calculated from the proportion of participants giving a positive score (5, 6, or 7 on a Likert-scale) to an item. Positive APSQ-score was defined as a mean score ≥ 4.50, neutral score in between 3.50 and 4.49, and negative score was < 3.50. The cut-off points were assigned based on the midpoint of the used Likert scale to facilitate classifying and reporting results in a uniform manner and were not implied in the statistical analysis. We took the author’s permission to use the validated questionnaire through email. The questionnaire was constructed and distributed in English language.

### Statistical analysis

Descriptive statistics were used to present participants’ demographics as frequencies and percentages. Students’ responses to each statement in the APSQ-III were presented as mean ± standard deviation. The overall APSQ-Score was calculated by dividing the sum of responses to each item by the total number of items for each respondent. This was used to describe the overall patient safety attitudes on a scale of 1 to 7 (as in Likert-scale). The overall APSQ-Score was represented as mean ± standard deviation for all participants and subgroups. Subgroup analysis was conducted using One-way ANOVA and Student’s t-test to assess associations between participants’ demographics and their APSQ-Score. The significance level was set at α of 0.05. Statistical analysis was done using the Statistical Package for the Social Sciences (Version 26.0, SPSS Inc., Chicago, IL, USA).

### Ethical considerations

This study was approved by the Institutional Review Board of Jordan University of Science and Technology (IRB Ref: 2023/157/32). Informed consent was obtained from all participants prior accessing the questionnaire. Measures were taken to ensure confidentiality and privacy of all participants. All methods were carried out in accordance with relevant guidelines and regulations.

## Results

### Demographics of participants

Our study included 1226 medical students from six different universities. The mean age of participants in years was 20.84 (SD ± 1.8) and the majority were females (n = 827; 67.5%). The distribution of our sample across universities and academic years was equivalent, indicating an equitable representation of participants within mentioned categories. Participants from the clinical stage were 50.9% (n = 624) and the pre-clinical stage were 49.1% (n = 602). First-generation medical students constituted 73.6% of our included sample. Participants’ demographics are displayed in Table 1.

**Table 1:** Participants’ Demographics

	N	%	Overall APSQ-Score Mean ± SD	*p–*value
**Total**	1226	100	4.9 ± 0.65	
**Gender**				
Male	399	67.5	4.9 ± 0.68	0.058
Female	827	32.5	5.0 ± 0.63	
**University**				
Al-Balqa’ Applied university	204	16.6	4.9 ± 0.75	
Hashemite university	207	16.9	4.9 ± 0.64	
Jordan university of science and technology	198	16.2	5.0 ± 0.54	0.013
Mutah university	204	16.6	4.9 ± 0.63	
University of Jordan	209	17.0	5.1 ± 0.61	
Yarmouk university	204	16.6	4.9 ± 0.70	
**Academic year**				
First year	206	16.8	4.9 ± 0.69	
Second year	198	16.2	4.9 ± 0.72	
Third year	198	16.2	4.9 ± 0.69	
Fourth year	215	17.5	4.9 ± 0.63	0.006
Fifth year	264	21.5	5.0 ± 0.57	
Final year	145	11.8	5.1 ± 0.58	
**First generation medical student?**				
Yes	902	73.6	5.0 ± 0.65	0.756
No	324	26.4	4.9 ± 0.66	

### Attitudes toward patient safety

The mean overall APSQ score was positive with a score of 4.9 (SD ± 0.65). Among the different domains, participants scored the highest score in “Working hours as error cause” with a mean value of 5.86 (SD ± 1.37) followed by “Team functioning” and “Importance of patient safety in the curriculum” with mean scores of 5.77 (SD ± 1.27) and 5.25 (SD ± 0.99), respectively. Neutral attitudes were reported only in “Professional incompetence as error cause” domain with a mean score of 3.56 (SD ± 0.69). No negative attitudes were found, Scores to all domains and items are shown in Table 2.

**Table 2:** Means and standard deviation of students’ responses to the Attitudes to Patient Safety Questionnaire III (APSQ-III)

Factor Items	Mean	SD	% Of positive responses
**Patient safety training received**	**4.92**	**1.365**	
1- My training is preparing me to understand the causes of medical errors	4.89	1.620	62.4
2- I have a good understanding of patient safety issues as a result of my undergraduate medical training.	4.85	1.529	62.3
3- My training is preparing me to prevent medical errors	5.03	1.603	67.8
**Error reporting confidence**	**4.89**	**1.466**	
4- I would feel comfortable reporting any errors I had made, no matter how serious the outcome had been for the patient	4.82	1.828	60.0
5- I would feel comfortable reporting any errors other people had made, no matter how serious the outcome had been for the patient.	4.73	1.677	57.9
6- I am confident I could talk openly to my supervisor about an error I had made if it had resulted in potential or actual harm to my patient	5.13	1.673	67.3
**Working hours as error cause**	**5.86**	**1.346**	
7- Shorter shifts for doctors will reduce medical errors	5.88	1.553	81.6
8- By not taking regular breaks during shifts doctors are at an increased risk of making errors	5.86	1.538	83.0
9- The number of hours doctors work increases the likelihood of making medical errors	5.85	1.508	82.5
**Error inevitability**	**5.17**	**1.133**	
10- Even the most experienced and competent doctors make errors	5.61	1.509	79.4
11- A true professional does not make mistakes or errors ^R^	4.99	1.737	63.8
12- Human error is inevitable	4.91	1.577	60.8
**Professional incompetence as error cause**	**3.56**	**0.694**	
13- Most medical errors result from careless nurses	3.74	1.522	29.7
14- If people paid more attention at work, medical errors would be avoided ^R^	2.80	1.354	11.2
15- Most medical errors result from careless doctors ^R^	3.76	1.517	28.9
16- Medical errors are a sign of incompetence ^R^	3.95	1.477	32.1
**Disclosure responsibility**	**4.75**	**0.886**	
17- It is not necessary to report errors which do not result in adverse outcomes for the patient ^R^	4.91	1.777	60.6
18- Doctors have a responsibility to disclose errors to patients only if they result in patient harm	3.83	1.759	34.0
19- All medical errors should be reported	5.52	1.626	75.0
**Team functioning**	**5.77**	**1.274**	
20- Better multi-disciplinary teamwork will reduce medical errors	5.72	1.387	82.0
21- Teaching teamwork skills will reduce medical errors	5.82	1.373	85.6
**Patient involvement in reducing error**	**5.03**	**1.314**	
22- Patients have an important role in preventing medical errors	4.79	1.535	60.8
23- Encouraging patients to be more involved in their care can help to reduce the risk of medical errors occurring	5.28	1.433	74.5
**Importance of patient safety in the curriculum**	**5.25**	**0.992**	
24- Teaching students about patient safety should be an important priority in medical students training	5.97	1.363	85.7
25- Patient safety issues cannot be taught and can only be learned by clinical experience when qualified ^R^	3.96	1.718	38.4
26- Learning about patient safety issues before I qualify will enable me to become a more effective doctor.	5.81	1.383	83.4

SD: Standard deviation. ^R^ indicates reverse coded items

Positive responses proportion to each item is shown in Table 2. Out of 26 items, 19 items had a positive response proportion ≥ 60%. Most positive responses were scored in the item “Teaching students about patient safety should be an important priority in medical students training” by 85.7%, followed by “Teaching teamwork skills will reduce medical errors” by 85.6% positive responses. However, the item “If people paid more attention at work, medical errors would be avoided (R)” scored the least by 11.2% positive responses.

### Participants’ demographics and attitudes to patient safety

Table 3. shows the association between gender and first-generation student status with the mean ASPQ scores to different domains. Females scored significantly higher than males in four domains: “Patient safety training received”, “Working hours as error cause”, “Error inevitability”, and “Professional incompetence as error cause” (p ≤ 0.05). Males scored significantly higher in one domain only “Patient involvement in reducing error” (p ≤ 0.05). Other domains showed no differences between genders. First generation medical students had a significantly lower score for “Professional incompetence as error cause” (p ≤ 0.05).

**Table 3:** Association between participants’ gender and first-generation student status with their APSQ score to each domain

Item Domains	Gender					FGM				
	Female		Male			No		Yes		
	Mean	SD	Mean	SD	*p*-value	Mean	SD	Mean	SD	*p*-value
Patient safety training received	4.96	1.33	4.84	1.44	0.007	4.89	1.43	4.94	1.34	0.183
Error reporting confidence	4.93	1.46	4.83	1.48	0.485	4.84	1.52	4.91	1.45	0.288
Working hours as error cause	5.95	1.31	5.68	1.41	0.003	5.84	1.40	5.87	1.33	0.341
Error inevitability	5.18	1.08	5.15	1.23	0.002	5.11	1.12	5.19	1.14	0.592
Professional incompetence as error cause	3.58	0.66	3.53	0.75	0.002	3.60	0.75	3.55	0.67	0.021
Disclosure responsibility	4.76	0.88	4.73	0.89	0.957	4.66	0.88	4.79	0.89	0.507
Team functioning	5.77	1.26	5.77	1.31	0.268	5.77	1.27	5.77	1.28	0.980
Patient involvement in reducing error	5.02	1.27	5.06	1.39	0.025	4.97	1.37	5.06	1.29	0.732
Importance of patient safety in the curriculum	5.26	0.96	5.22	1.06	0.047	5.21	1.00	5.26	0.99	0.803

SD: Standard deviation, FGM: First-generation medical student

Clinical stage students scored significantly higher in “Team functioning”, “Error inevitability” and “Working hours as error cause” domains (p ≤ 0.05). Interestingly, pre-clinical students recorded more positive attitudes in “Patient safety training received” and “Disclosure responsibility” domains. The association between participants’ academic stage and APSQ score to each domain is shown in Table 4.

**Table 4:** Association between participants’ academic stage and APSQ score to each domain

Item Domains	Academic Stage				
	Pre-clinical Stage		Clinical Stage		
	Mean	SD	Mean	SD	*p*-value
Patient safety training received	5.02	1.43	4.83	1.29	0.015
Error reporting confidence	5.00	1.44	4.80	1.49	0.630
Working hours as error cause	5.59	1.42	6.12	1.21	< 0.001
Error inevitability	5.00	1.19	5.34	1.05	0.010
Professional incompetence as error cause	3.52	0.70	3.60	0.68	0.287
Disclosure responsibility	4.77	0.93	4.73	0.84	0.034
Team functioning	5.57	1.36	5.96	1.16	< 0.001
Patient involvement in reducing error	4.97	1.36	5.09	1.27	0.140
Importance of patient safety in the curriculum	5.17	1.03	5.32	0.95	0.067

SD: Standard deviation

## Discussion

According to estimates from the WHO, an unacceptable number of individuals die each year as a result of unsafe medical care [1]. It is commonly reported that one out of every ten hospitalized patients suffer injury, at least half of which might have been avoided [1]. For example, medical mistakes are the third leading cause of mortality in the United States [3]. Unfortunately, a lot of students lack the self-confidence to report medical errors they or others have made. Yet, disclosing errors enhances the standard of treatment for new patients [7–9]. The importance of reporting medical errors is to enhance awareness and prevent recurrence, it is important to learn from the reasons of past accidents and near-misses by attempting to understand why they have occurred in the first place and then figure out how to avoid them [10, 11]. Our study provides a base for future research and emphasizes the value of educating medical students in universities about patient safety.

The positive overall attitudes displayed by medical students in our study towards patient safety concepts are encouraging. These findings indicate that medical students are receptive to the principles and practices that promote patient safety.

Among the different domains assessed, the most positive attitudes were observed in the domain of “Working hours as error cause.“ This finding reflects an understanding that excessive workload and fatigue can contribute to errors and compromise patient care. This awareness is crucial as it highlights the importance of effective scheduling and promoting work-life balance to mitigate the risks associated with extended work hours.

Another domain that received high positive attitudes was “Team functioning.“ This indicates that medical students value the role of teamwork among healthcare professionals. Recognizing that effective collaboration and communication can prevent errors and improve patient outcome. Furthermore, the positive attitudes towards the “Importance of patient safety in the curriculum” domain highlight that medical students acknowledge the need for patient safety education to be an integral part of their training. They recognize the importance of incorporating patient safety competencies into medical curricula is crucial for providing high-quality care and minimizing harm to patients. On the other hand, Neutral attitudes were reported in only one domain “Professional incompetence as error cause”.

We investigated the association between patient safety attitudes and first-generation students’ status as they bring unique perspectives and diverse backgrounds to medical education, shedding light on their potential roles in addressing health disparities and shaping the future of patient-centered care. First-generation medical students scored significantly lower score in “Professional incompetence as error cause” domain. This could be attributed to not having a good exposure to the medical profession and they may not fully understand the complexity and nuances of the healthcare system.

In Jordan, the academic program is divided into two halves, the first three years are the pre-clinical stage where students are taught basic subjects on the university campus and are not involved in patient care, and the later three years are the clinical stage where students are directly involved in patient care. The clinical training is done in university hospitals and at local hospitals, affiliated with Jordan Ministry of Health or Royal Medical Services; thus students from different universities sometimes share the same training hospital and attendings, making their experience and exposure similar during their clinical training.

The differences observed in attitudes towards patient safety domains between students in the clinical stage and those in the pre-clinical stage provide valuable insights into the evolving perspectives of medical students as they progress through their education.

Students in the clinical stage demonstrated more favorable attitudes towards “Team Functioning,“ “Error Inevitability,“ and the idea that “Working Hours” can contribute to errors. This finding suggests that clinical students have more hands-on experience during rotations and patient care so they develop a greater appreciation for the importance of teamwork. The higher scores in the domain of “Error Inevitability” could be attributed to their exposure to real-life medical situations, where they witness the complex nature of healthcare delivery. This may lead to a recognition that errors are not entirely preventable.

Moreover, the finding that students in the clinical stage scored higher in the domain of “Working Hours” as a contributing factor to errors suggests that they are aware of the potential risks associated with long working hours. This aligns with research and concerns about the impact of fatigue and excessive workload on healthcare professionals’ performance and patient safety [23, 24].

In contrast, students in the pre-clinical stage scored significantly higher in the domains of “Patient Safety Training Received” and “Disclosure Responsibility.“ These findings suggest that pre-clinical students recognize the need for a solid foundation in patient safety concepts and skills to ensure safe and effective care delivery. A study that examined patients’ and physicians’ attitudes regarding the disclosure of medical errors found that physicians often avoid explicitly stating the occurrence of errors and struggle to provide emotional support [25].

Other studies have assessed medical students’ attitudes toward patient safety using the APSQ [26–35]. The domain that received the highest score in our study, “working hours as an error cause”, was consistent with other studies among medical students in Tunisia, Singapore, and Hong Kong [32, 35]. Long working hours were found to be an important risk factor for burnout among residents which in turn is a major cause of medical errors [36, 37]. A cross-sectional study in 2021 that aimed to determine the prevalence of burnout among resident physicians in Jordan showed that 77.5% were found to have burnout [37]. Medical students are constantly in contact with residents leading to increased awareness of working hours as a cause of medical errors. A recent systematic review and meta-analysis that aimed to study the relationship between long working hours and accidents and injuries, found that weekly working hours > 55 h were associated with an increased risk of incidents [38]. To reduce burnout among healthcare providers we suggest that institutions prioritize their well-being, provide flexible schedules, encourage regular breaks, promote work-life balance, and provide stress management resources to achieve better healthcare quality.

“Team functioning” domain received positive overall attitudes, which was consistent with previous study findings [32, 33, 35, 39]. Medical students may have chosen team functioning as an important factor in preventing medical errors for several reasons. Firstly, they are learning about the importance of multi-disciplinary teams and effective teamwork in providing high-quality patient care due to the complexity of healthcare management. Secondly, clinical stage students may have observed the negative impact of poor team functioning on the quality of health care provided.

In accordance with other studies, we found that females scored statistically significant positive attitudes in “Professional incompetence as error cause” domain [25, 27]. Other studies reported male students having more confidence in reporting errors [25, 27]. We did not find any association between gender and error reporting confidence. Those differences between genders and patient safety domains can be due to differences in socialization, cultural norms, and personal experiences. Further research with larger and more diverse samples would be needed to establish a more robust understanding of gender differences in regard to patient safety concepts.

Strengths of our study include the large sample size that comprises students from all medical schools with equal distribution between demographics, therefore can be regarded as representative of medical students in Jordan. Also, we used a validated questionnaire developed specifically for medical students with good reliability coefficients (0.64–0.82). The findings of our study provide baseline data for patient safety attitudes among medical students, on which further educational program improvements can be built. Limitations of our study include the questionnaire-based study design which is subject to recall bias. Besides, we did not collect background data for participants involved in our study, which may influence their attitudes, and we did not conduct regression analysis models because it was difficult to control confounding variables. It is worth mentioning that our findings reflect self-reported data, thus may not accurately reflect the true behaviors of medical students.

Further research studies are needed to evaluate real students’ behaviors and attitudes. Future studies could use a combination of self-reported data and objective measures, such as observations or simulations, to assess the attitudes and behaviors of medical students toward patient safety concepts. This would provide a more comprehensive understanding of the attitudes of medical students and help validate their self-reported data. Additionally, follow-up studies that track the attitudes and behaviors of medical students over time would also provide valuable insights into the stability and reliability of the attitudes they reported. Healthcare organizations are recognizing the significance of changing their organizational culture to enhance patient safety. As interest in safety culture grows, there is a demand for assessment tools that specifically address the cultural aspects of patient safety improvement initiatives [40].

## Conclusions

Undergraduate medical students in Jordan showed positive overall attitudes toward patient safety concepts. Neutral attitudes were reported only in the “Professional incompetence as error cause” domain, while no domain received a negative attitude score. Gender, first-generation student status and academic year differences had a significant association with patient safety domains. Our findings indicate a promising future for patient safety culture within the healthcare system with improved patient outcomes. Medical schools are recommended to continue to prioritize and enhance patient safety education throughout their curriculum; by integrating these concepts into lectures, case-based discussions, and clinical rotations. This study provides baseline data to improve current educational programs and enhance a patient safety culture among medical students. Further studies are warranted to investigate the real behaviors toward patients’ safety and the impact of educational programs in developing this culture, as our study was based on self-reported data.
